# *Allium sphaeronixum* (Amaryllidaceae), A New Species from Turkey

**DOI:** 10.3390/plants12112074

**Published:** 2023-05-23

**Authors:** Mine Koçyiğit, Cristina Salmeri, Neriman Özhatay, Erdal Kaya, Salvatore Brullo

**Affiliations:** 1 Department of Pharmaceutical Botany, Faculty of Pharmacy, Istanbul University, Beyazıt, TR-34452 Istanbul, Turkey; mkocyigit@istanbul.edu.tr (M.K.); neriman.ozhatay@emu.edu.tr (N.Ö.); 2 Department of Biological, Chemical and Pharmaceutical Sciences and Technologies (STEBICEF), University of Palermo, Via Archirafi 38, 90123 Palermo, Italy; 3 NBFC, National Biodiversity Future Center, Piazza Marina 61 (c/o palazzo Steri), 90133 Palermo, Italy; 4 Faculty of Pharmacy, Doğu Akdeniz Üniversitesi, 98628 Famagusta, Cyprus; 5 Atatürk Central Horticultural Research Institute, 77102 Yalova, Turkey; erdal_kaya@msn.com; 6 Department of Biological, Geological and Environmental Sciences, University of Catania, Via A. Longo 19, 95125 Catania, Italy; salvo.brullo@gmail.com

**Keywords:** Anatolia, endemic, sect. *Codonoprasum*, karyology, leaf anatomy, taxonomy, seed micromorphology

## Abstract

In this paper, *Allium sphaeronixum*, a new species of the sect. *Codonoprasum* from Turkey, is described and illustrated. The new species is endemic to Central Anatolia, limited to the area of Nevşehir, where it grows on sandy or rocky soil at an elevation of 1000–1300 m a.s.l. Its morphology, phenology, karyology, leaf anatomy, seed testa micromorphology, chorology, and conservation status are examined in detail. The taxonomic relationships with the closest allied species, *A. staticiforme* and *A. myrianthum*, are also highlighted and discussed.

## 1. Introduction

*Allium* L. is the largest genus of petaloid monocotyledons, comprising over 1100 accepted taxa [1]. It is represented by about 230 taxa in Turkey, grouped into 14 sections, with 86 endemics. In particular, the sect. *Codonoprasum* Rchb. is the second largest section in Turkey, with ca. 60 taxa, of which more than 30 are endemic [2,3,4].

Many taxonomical studies on this section have been carried out over the last years, resulting in the reassessment of some critical taxa and the description of many new endemic species from Turkey, e.g., [2,3,4,5,6,7,8,9,10,11].

During field surveys in the neighborhood of Nevşehir (Central Anatolia, Turkey), some populations of a very peculiar *Allium* clearly belonging to the sect. *Codonoprasum* were collected and investigated. Morphologically, these plants showed some resemblance with *A. myrianthum* Boiss., a species widespread in Anatolia, as well as with *A. staticiforme* Sm., found in the Aegean islands. According to [12], these species belong to a very peculiar group, with *A. staticiforme* as the most representative taxon, which is distributed in the East Mediterranean territories. The *A. staticiforme* group is well differentiated from all other known taxa of sect. *Codonoprasum* by having the following distinct selection of morphological traits: dense spherical inflorescence, with rather short spathe valves; small-sized perigon (2–4 mm long); stamen filaments, with all or at least the inner ones exserted; and seeds less than 4 mm long.

The aim of this study was to perform careful biosystematic analyses using living specimens cultivated both in the Botanical Garden of Catania (Italy) and in the Geophyte Garden in Yalova (Turkey) in order to clarify the taxonomic position of Nevşehir plants and their relationships within the *staticiforme* group. The performed investigations revealed that the populations from Nevşehir were closely related to *A. myrianthum* and *A. staticiforme*, which indeed clearly differ in many significant morphological features regarding the scape, number, and size of leaves; size and/or color of flower pieces (tepals, anthers, ovary, and style); and fruit size. A relevant diversity was also found in the karyotype structure, leaf anatomy, and seed microsculptures. Therefore, this *Allium* coming from Nevşehir is here treated as a new species for science, named *A. sphaeronixum*.

## 2. Results

### 2.1. Taxonomy

**Figure 1:**
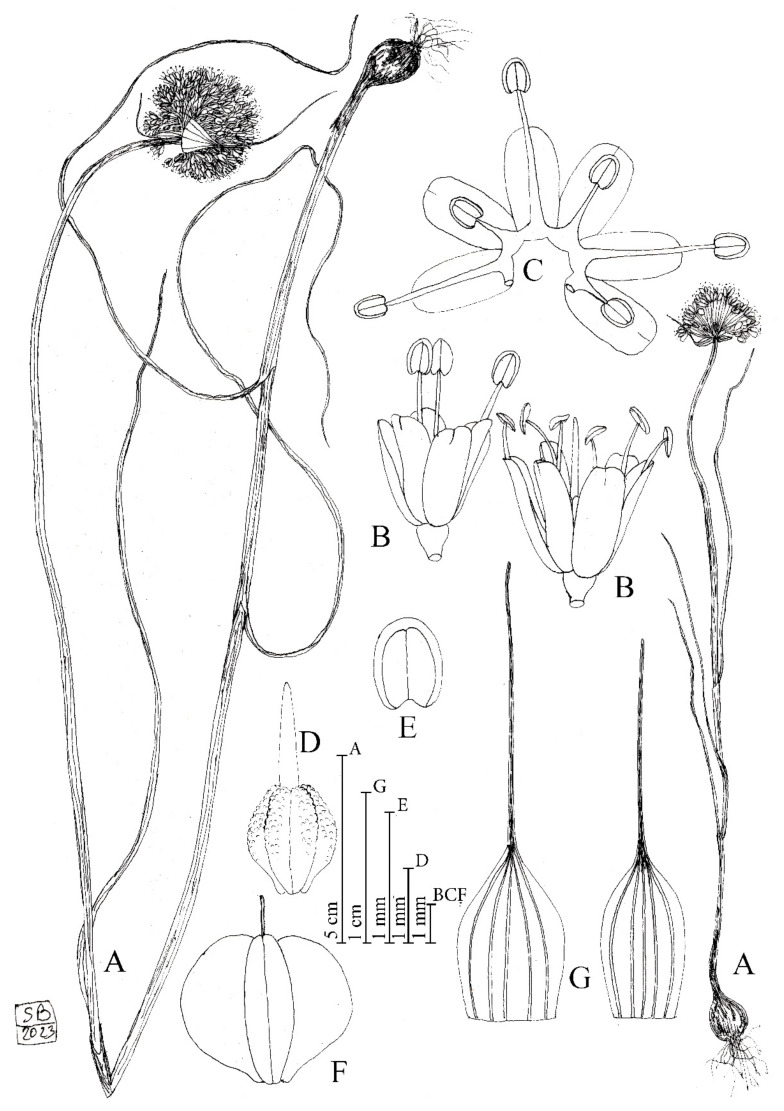
Diagnostic features of *Allium sphaeronixum*. (A) Habit. (B) Flowers. (C) Open perigon and stamens. (D) Ovary. (E) Anther. (F) Capsule. (G) Spathe valves. Illustration by S. Brullo based on living material from the type locality.

**Figure 2:**
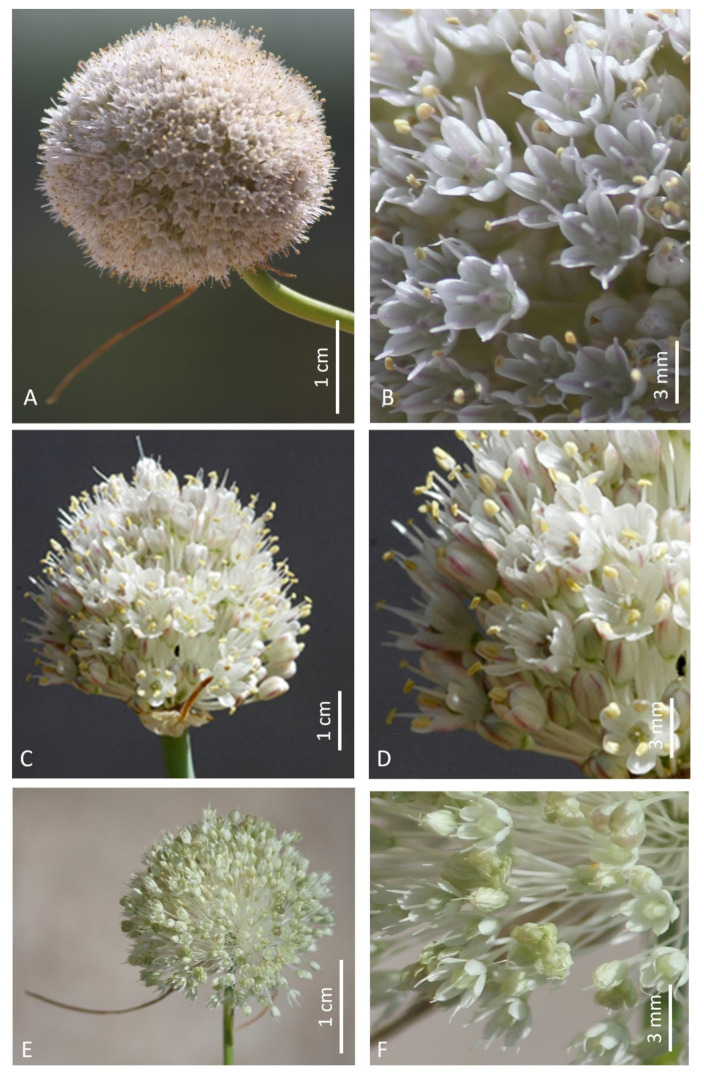
Inflorescences and flower details of *A. sphaeronixum* (**A**,**B**), *A. staticiforme* (**C**,**D**), and *A. myrianthum* (**E**,**F**).

*Allium sphaeronixum* Koçyiğit, Salmeri, Özhatay, Kaya and Brullo *sp. nov.* (Figure 1, Figure 2 and Figure S1, Table 1).

Type: TURKEY: Central Anatolia, Nevşehir, colline sabbiose, 30 June 1988, *S. Brullo*, *P. Pavone* and *P. Signorello s.n.* (holotype: CAT!; isotypes: CAT!, ISTE!).

Diagnosis: Allio myriantho similis, sed scapo ad 1/2 longitudinem vaginis foliorum tecto, foliis 3-4, lamina latiore, valvis spathae inaequalibus, tepalis albo-roseis, subaequalibus, longioribus, filamentis staminorum longioribus, antheris brevioribus, ovario breviter stipitate, longiore, stylo longiore, capsula maiore.

Description: Perennial plant. *Bulb* ovoid, 1–2 × 0.8–1.5 (–2) cm; outer tunics grey-brown, fibrous-coriaceous, inner ones white, membranous. *Scape* glabrous, erect, 20–80 cm tall, cylindrical, glaucous, covered for 1/2 of its length by the leaf sheaths. *Leaves* 3–4, with blade 2–3 mm wide, up to 20 cm long, subcylindrical, ribbed, fistulous, and glabrous. *Spathe* persistent, with two appendiculate unequal valves, the largest 5 (–7)-nerved and 2.5–4 (–5.5) cm long, subequal to longer than the inflorescence, the smallest (3–) 5-nerved and 1.5–3 (–3.5) cm long, shorter or slightly longer than the inflorescence. *Inflorescence* dense, globose, (1.5–) 2.5–3 (–4) cm in diameter; pedicels subequal, (0.8–) 1–1.5 (–2) cm long. *Perigon* campanulate; tepals subequal, milky white suffused with pink, oblong-elliptic, rounded at apex, (2.5–) 2.8–3.5 mm long, inner tepals 1.1–1.3 mm wide, outer tepals 1.4–1.5 mm wide. *Stamen filaments* simple, white, sometimes tinged with pink, the outers 1.2–2 mm long, at first shorter than perigon, then exserted, the inners always exserted from the perigon 3–4 mm long, connate below into an annulus 0.3–0.4 mm high. *Anthers* yellow-tinged with purple, oblong, rounded at apex, 0.7–0.9 × 0.5–0.6 mm. *Ovary* obovoid, stipitate at base, green, slightly tuberculate, 1.8–2 × 1.4–1.5 mm. *Style* pinkish-white, 1.9–2 mm long. *Capsule* green, subglobose to globose-obovoid, 3.8– 4 × 3.8– 4 mm. *Seeds* 3.5–3.7 × 2–2.2 mm, outline semi-ovoid. 

Etymology: The specific epithet, coming from the Latin words *sphaera* = ball and *nix* = snow, refers to the globose whitish inflorescence resembling a snowball (Figure 2A). 

Suggested Turkish name: The Turkish name of this species is suggested as ‘Kartopu Soğanı’ [13].

Phenology: The species flowers from July to mid-August, and fruit ripening occurs ca. one month after flowering.

### 2.2. Karyology

**Figure 3:**
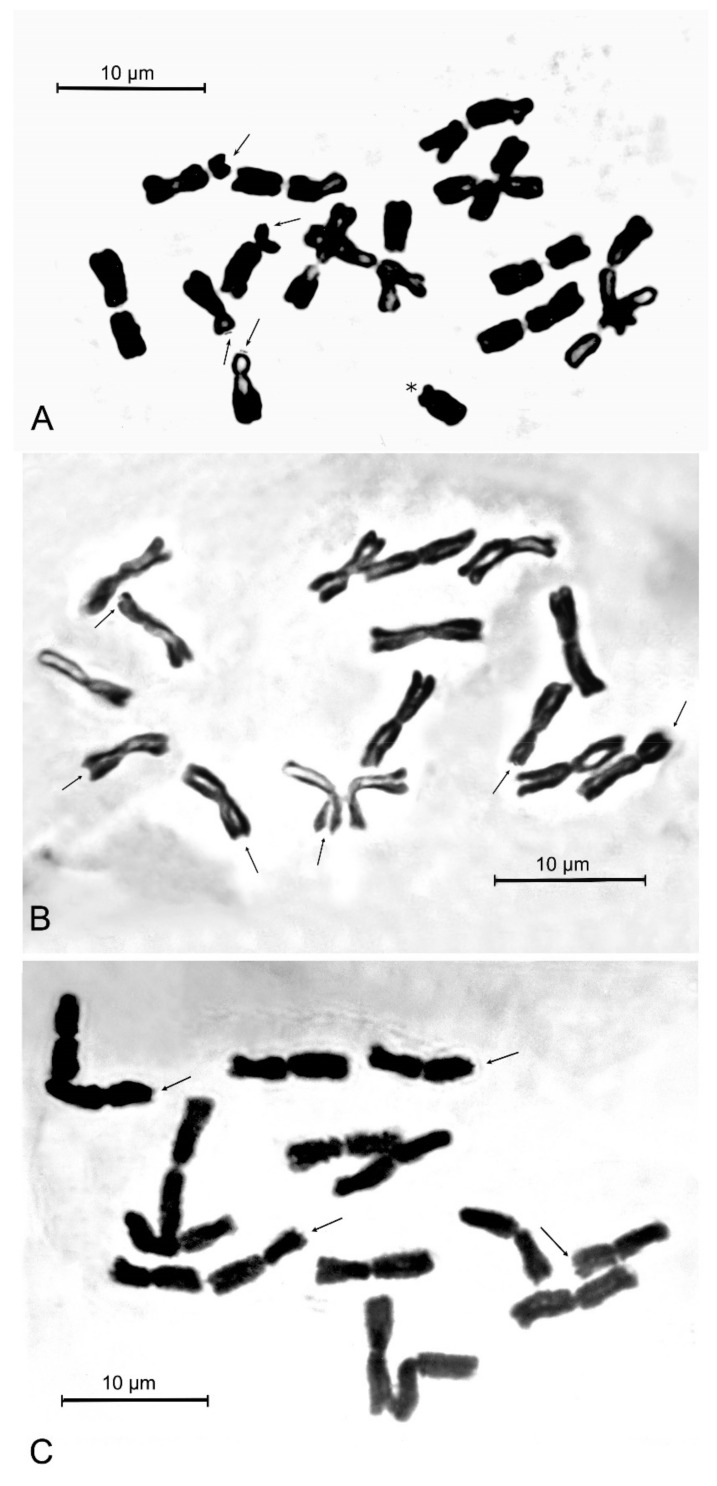
Mitotic metaphase plates of the new species and its closest related taxa from respective type localities. (**A**) *Allium sphaeronixum* (Nevşehir, TK); (**B**) *A. myrianthum* (Pamukkale, TK); (**C**) *A. staticiforme* (Kimolos is., Goupa, GR). Arrows indicate satellited chromosomes, asterisk indicates B-chromosome.

**Figure 4:**
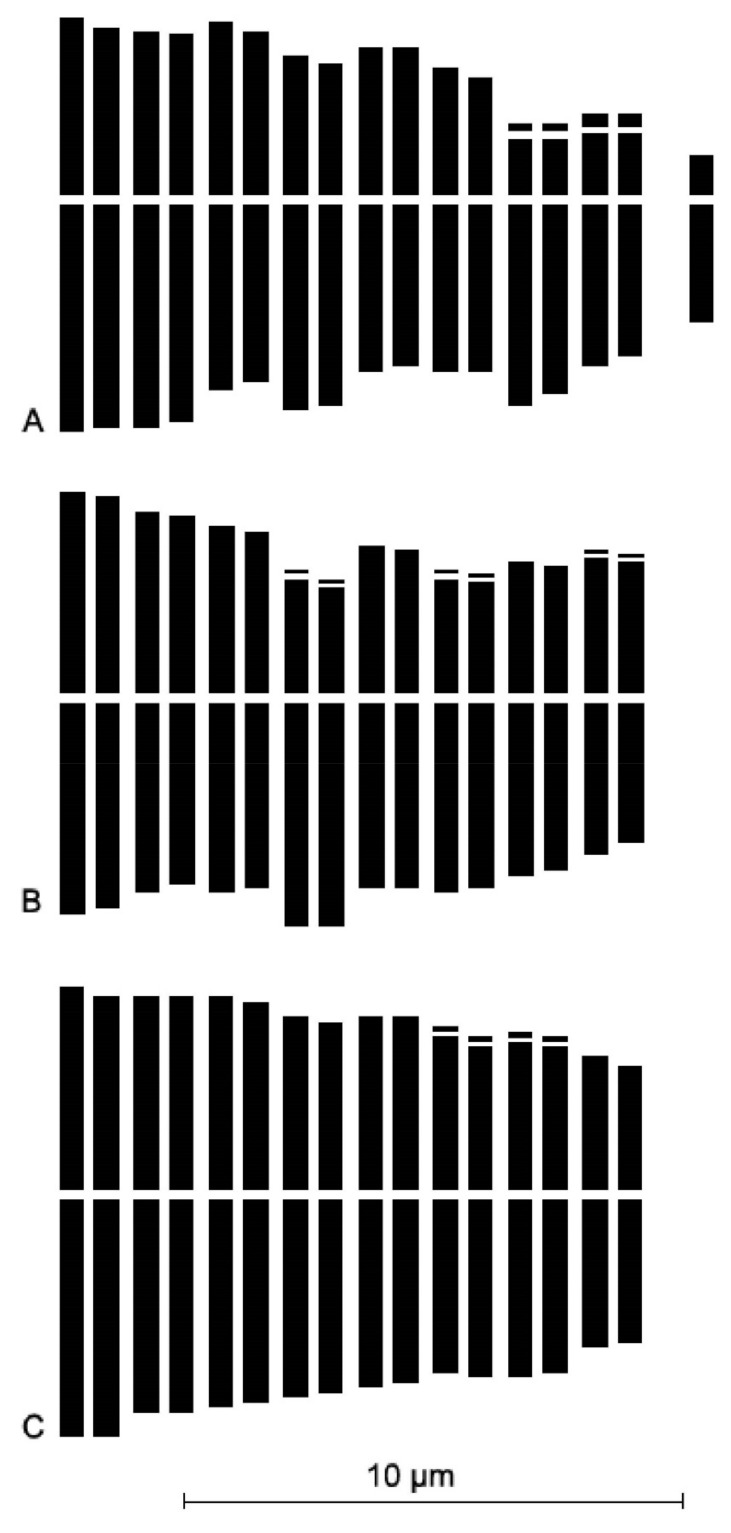
Karyograms of the new species and its closest related taxa from respective type localities. (**A**) *Allium sphaeronixum*; (**B**) *A. myrianthum*; (**C**) *A. staticiforme*.

The somatic chromosome number of *Allium sphaeronixum* (Figure 3A) was found to be 2*n* = 2*x* = 16 in all studied samples. The karyotype is rather asymmetrical, comprising only three fully metacentric pairs, while the remaining chromosomes include three meta-submetacentric pairs (arm ratio exceeding 1.30), one submetacentric and one subtelocentric pair, the last two pairs being, respectively, macro- and micro-satellited on the short arms (Figure 4A). One small B-chromosome was detected in most of the observed metaphase plates. Thus, the chromosome formula of *A. sphaeronixum* can be expressed as 2*n* = 2*x* = 16 = 6 m + 6 msm + 2 sm^sat^ + 2 st^sat^ + 0-1B. The absolute chromosome length varied from 9.88 ± 0.6 μm of the longest chromosome to 5.31 ± 0.4 μm of the shortest one, with a mean chromosome length of 7.75 ± 1.4 μm. The relative chromosome length varied from 7.97% ± 0.3 to 4.28% ± 0.3. The arm index varied on average from 1.08 to 3.39, while the centromeric index ranged from 48.11 to 22.66, with a mean value of 39.3 ± 8.7.

As far as the two most allied species are concerned, the chromosome complements of both *Allium myrianthum* and *A. staticiforme* from their respective type localities were also examined for comparison (Figure 3B,C). The karyotypes of these species appeared quite different from that of *A. sphaeronixum*, though sharing the same diploid (2*n* = 16) chromosome arrangement [12]. Karyotypes of *A. staticiforme* mostly showed more or less median chromosomes (five pairs fully metacentric and two pairs meta-submetacentric), while two chromosomes were submetacentric and microsatellited on the short arm, which can be determined by the following chromosome formula: 2*n* = 2*x* = 16: 8 m + 2 m^sat^ + 2 msms + 2 msm^sat^ + 2 sm^sat^ (Figure 4C). Conversely, *A. myrianthum* revealed a quite homogeneous karyotype, characterized by only metacentric chromosomes, two pairs of which were microsatellited on the short arm, with a chromosome formula 2*n* = 2*x* =16: 12 m + 4 m^sat^ (Figure 4B). All karyomorphometric parameters for the new species and its closest allied taxa are given in Table 2.

### 2.3. Leaf Anatomy

**Figure 5:**
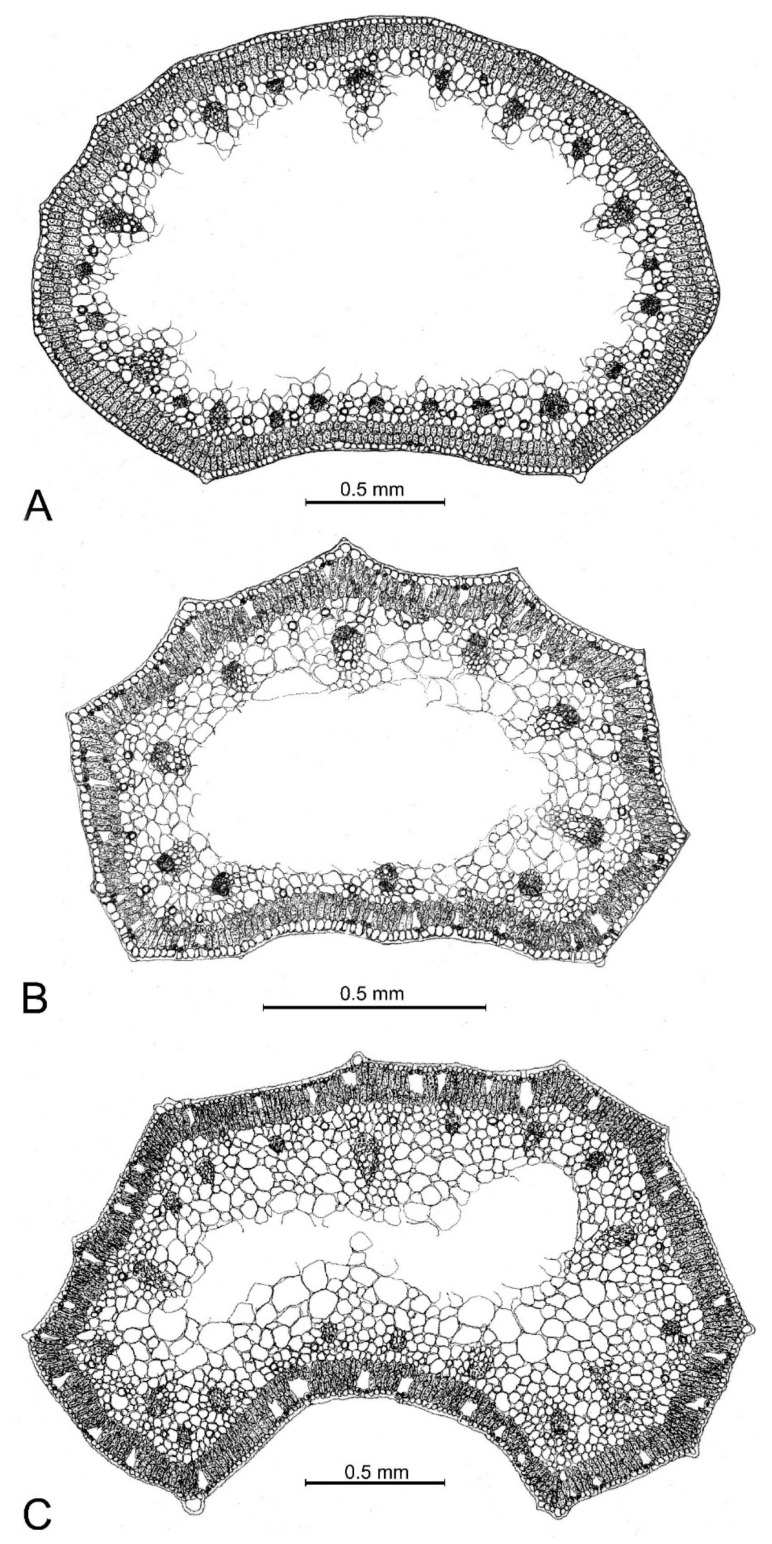
Leaf cross-sections of the new species and its closest related taxa from respective type localities. (**A**) *Allium sphaeronixum*; (**B**) *A. myrianthum*; (**C**) *A. staticiforme*.

The leaf cross-section of *Allium sphaeronixum* showed a subcylindrical outline, with several inconspicuous ribs along the abaxial surface, while the adaxial one is flat to slightly concave and bordered by two evident ribs. The epidermis has small cells covered by a thin cuticle. Stomata are numerous and distributed along the whole leaf perimeter. The palisade tissue is compact and uniformly arranged in two layers of cylindrical cells, a little smaller in the adaxial face. The spongy tissue appeared distributed only in the peripherical part of the mesophyll, as the leaf is widely fistulous. Many secretory ducts occurred under the palisade tissue. There were about 8 big-sized vascular bundles in the abaxial part, alternating with. ca. 10 smaller ones, while 5 small vascular bundles occurred along the adaxial face (Figure 5A, Figure S2A and Figure S3A).

Conversely, the leaf cross sections of *A. myrianthum* (Figure 5B, Figure S2B and Figure S3B) showed a semicylindrical outline with eight regularly prominent ribs with distinct hyaline tips. The epidermis consisted of large cells with a well-developed cuticle layer. The palisade tissue appeared two-layered, while the spongy tissue occurred only in the peripheral part because the mesophyll appeared widely fistulous in the center. Seven large vascular bundles occurred along the abaxial faces in correspondence with the ribs, while there were just three vascular bundles along the adaxial face. 

As in *A. myrianthum*, the leaves in *A. staticiforme* also showed a semicylindrical outline (Figure 5C, Figure S2C and Figure S3C), with 10–13 more or less prominent ribs along the abaxial surface, each ending with a small hyaline tip, while the adaxial face was smooth and distinctly concave. The epidermis showed small cells covered by a well-developed cuticle; the palisade tissue consisted of two layers of long cylindrical cells, while the spongy tissue appeared compact with small cells in the peripheral part, becoming looser with large cells in the center. There were 6–8 large-sized vascular bundles along the abaxial face alternating with 6 smaller ones, while only 5 small vascular bundles occurred in the adaxial face.

### 2.4. Seed Morphology and Micro-Sculpturing

The seed coat microstructures in *Allium* provide significant information for taxonomic treatment at a specific or sectional level because they represent a stable and conservative character [14,15,16,17,18,19,20,21,22,23,24,25,26].

**Figure 6:**
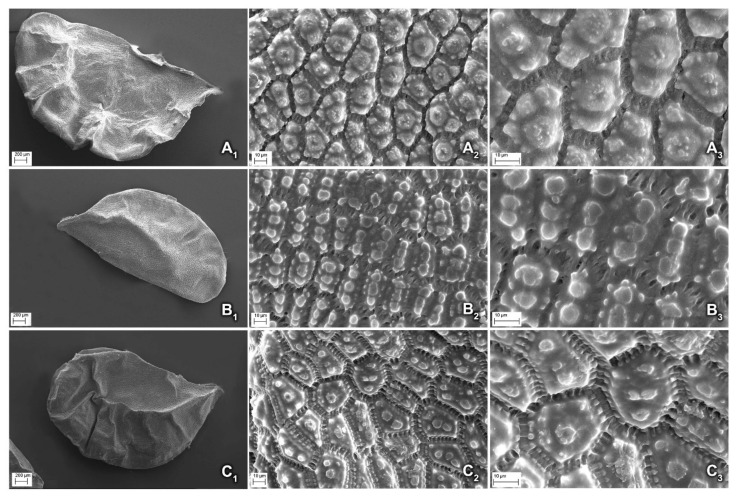
SEM micrographs of seed testa of the new species and its closest related taxa. (**A_1_**–**A_3_** ) *Allium sphaeronixum*; (**B_1_**–**B_3_** ) *A. myrianthum*; (**C_1_**–**C_3_** ) *A. staticiforme* (magnification: **A_1_**–**C_1_** = ×30; **A_2_**–**C_2_** = ×600; **A_3_**–**C_3_** = ×1200).

Overall, the seed testa micromorphology of *A. sphaeronixum* (Figure 6A) reflected the main pattern already detected in other species of the sect. *Codonoprasum* [9,19,21,23,27]. The seeds were 3.5–3.7 × 2–2.2 mm in size, showing a semi-ovoid outline and a minutely papillate surface. At high magnifications (600× and 1200×), the testa cells revealed a subpolygonal and nearly isodiametric shape (21–36.5 × 16.5–32 μm), with minutely and irregularly undulate borders. The anticlinal walls appeared rather depressed and partly covered by the presence of feebly strip-like depositions forming an intercellular region 1.8–6 μm wide. The periclinal walls were slightly raised, usually provided with a large central and papillate verruca, surrounded by smaller peripheral ones, which made them very variable in number and size.

Conversely, the seeds of *A. myrianthum* (Figure 6B) were 2.9–3.1 × 1.5–1.6 mm in size, characterized by subrectangular testa cells, 24.5–45.5 × 7–18.5 μm, with irregular undulate borders. The anticlinal walls were poorly detectable, appearing somehow flat to slightly channeled and covered by prominent strip-like connections forming a large intercellular region 8–12 μm wide. The periclinal walls were slightly raised, provided with 2–4 (–5) irregular and smooth verrucae arranged in a central row along the main cell axis, sometimes with some additional smaller and marginal verrucae.

As far as *A. staticiforme* is concerned, seeds (Figure 6C) were 2.9–3.1 × 1.8–1.9 mm in size, with irregularly polygonal cells, varying from sub-isodiametric to elongated (24.5–52 × 12.5–23.5 µm). The anticlinal walls were slightly channeled with Ω-like undulations connected through well-distinct strip-like depositions forming an intercellular region 8–10 μm wide. The periclinal walls were rather flat, with 1–3 median large verrucae further covered by small papillae, seldom with few small marginal verrucae.

### 2.5. Distribution and Ecology

**Figure 7:**
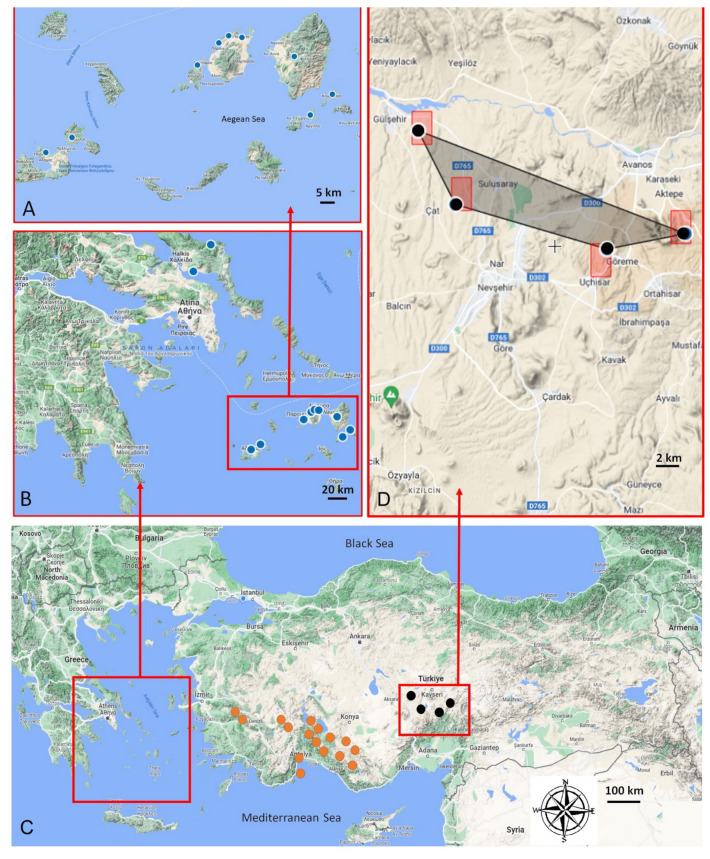
Geographic distribution of *A. sphaeronixum* and its closest allied *A. myrianthum* and *A. staticiforme*. (**A**,**B**) examined populations of *A. staticiforme* (blue), (**C**) *A. myrianthum* (orange), and *A. sphaeronixum* (black). (**D**) Area of occupancy of *A. sphaeronixum* with its four known populations.

The geographic distribution of *A. sphaeronixum* and its closest allied *A. myrianthum* and *A. staticiforme* is quite far from each other. While *A. staticiforme* is distributed in the Central Aegean Islands (Figure 7A,B), *A. myrianthum* grows in southwestern Anatolia (Figure 7C). This new species is localized near Nevşehir in Central Anatolia (Figure 7C,D), where it grows in maquis and dwarf shrub communities, on sandy or rocky substrata, at 1000–1300 m of elevation. Conversely, *A. myrianthum* usually grows in grasslands linked to more or less damp soils, while *A. staticiforme* can be found in the clearings of coastal garrigues or maquis on various types of substrata, from sea level up to an elevation of ca. 500 m.

### 2.6. Conservation Status

*Allium sphaeronixum* is currently known only from four populations of Turkey (Figure 7D), within an estimated area of occupancy (AOO) of 16.00 km^2^ and an extent of occurrence (EOO) of 58.59 km^2^. Fairy chimneys are a unique geological formation found in the Nevşehir province of Turkey. These tall, cone-shaped rock formations are formed from soft volcanic ash and tuff, which has been eroded over time by wind and water. The harder rocks on top of the fairy chimneys protect the softer rock beneath, creating a chimney-like shape. Fairy chimneys in Nevşehir are especially famous for their historical and cultural significance. In the past, people used these chimneys as dwellings, and some were even decorated with intricate carvings and frescoes. Today, many of these chimneys have been converted into hotels and tourist attractions. There is intense tourism pressure in the region. Therefore, following the criteria established by IUCN [28], an assessment of ‘Endangered’ (EN, criteria A4, B1abii, iii) is suggested for the new species.

### 2.7. Additional Examined Species

*Allium sphaeronixum* (paratypes): TURKEY. Nevşehir Province: Göreme, 9 August 1989, *S. Brullo* and *Signorello s.n.* (CAT); above Göreme, dry slopes, 1260 m, 21 July 1981, *N. Özhatay s.n.* (ISTE 47113); ibid. (ISTE 47120); near Gülşehir, 1000 m, 20 July 1981; between Nevşehir and Gülşehir, 1072 m, 7 July 2012, *E. Kaya 3920*; between Avanos and Ürgüp, near Zelve, 1065 m, 7 July 2012, *E.Kaya 3916*.

*A. myrianthum*: TURKEY. Denizli Province: Pamukkale, 02 July 1983, *A. H. Meriçli s.n.* (ISTE 50570); ibid., 24 June 1988, *S. Brullo*, *P. Pavone*, and *P. Signorello s.n.* (CAT); ibid., 21 June 1998, *S. Brullo* and *P. Pavone s.n.* (CAT); ibid., 24 July 2008, *M. Koçyiğit 204* (ISTE 87705); Acıgöl, 27 June 1987, *S. Brullo*, *P. Pavone*, and *P. Signorello s.n.* (CAT); ad Acıgöl, July 1987, *S. Brullo s.n.* (CAT); Acıgöl, Bozkurt, 23 June 1988, *S. Brullo*, *P. Pavone*, and *P. Signorello s.n.* (CAT); Marais de Méandre sous Hazli, s.d., *Boissier s.n.* (G-BOISS); humidis thermarum Hierapolis, June, *Boissier s.n.* (G-BOISS); Hierapolis in paludosis, June 1842, *Boissier s.n.* (FI-WEBB, G-BOISS); around Hotel Koru, 3 June 1978, *F. İlisulu s.n.* (AEF 6577). Izmir Province: plage sablonneuse à l’Est des salines de Smyrne, 3 June 1854, Balansa 390 (BM, FI-WEBB, G-BOISS); Ephesus, 31 May1972, *A.; T. Baytop s.n.* (ISTE 22098); Pyrenean, 30 May 1972, *N.; M. Tanker s.n.* (AEF 3586). Antalya Province: Near Zerk, 800 m, 25 June 1980, *Y. Ayaşlıgil 463* (ISTE 52465); Burmahan, east of Beşkonak, 950 m, 16 June 1982, *Y. Ayaşlıgil 826/5* (ISTE 52469); Çakları, Çalbalı Mountain, 2 km from Saklıkent, 1840 m, 02 June 1988, *N.; E. Özhatay s.n.* (ISTE 58974); Termassus, 800 m, 29 May 1990, *T. Baytop s.n.* (ISTE 61452); Between Alanya and Manavgat, Alarahan castle, 11 May 1979, *T. Baytop*, *B. Mathew*, *N. Sütlüpınar s.n.* (ISTE 41930); W Anatolia, Pamphilia, Alanya Saltschukishe Burg c. 150 m, 1 June 1988, Hertel 34128 (M); Serik, Turban-Belek facilities, 23 August 1993, *M. Koyuncu 10661* (AEF 17938); Kemer-Ovacık, Tahtalı Mountain, 4 km from Ovacık, 1030 m, 3 June 1988, *N.; E. Özhatay s.n.* (ISTE 59022). Burdur Province: Kızılkaya, 25 May 1966, *A. Baytop s.n.* (ISTE 9542). Isparta Province: Egirdir, Kenar Baglar, 01 May 1955, *A.; T. Baytop s.n.* (ISTE 4327); Kasnak Forest, 1500 m, 23 May 1982, *E. Şaner*, *M. Yemen*, *M. Coşkun 635* (AEF 10385); Anamas, above Oruçgazi Gedigi, 1650–2170 m, 07 July 1974, *H. Peşmen*, *A. Güner 1947* (ISTE 34282); Anamas, above Yenice, 1200–1400 m, 03 July 1976, *M. Koyuncu s.n.* (AEF 5613); around Zindan Cave, 1000 m, 24 June 2009, *M. Koçyiğit 271* (ISTE 87748); Eğirdir, below Mahmatlar village, 927 m, 23 July 2008, *M. Koçyiğit 196* (ISTE 87696); 900 m, 2 August 1976, *M. Koyuncu s.n.* (AEF 5577); around Kovada National Park, 910 m, 27 June 2009, *M. Koçyiğit 274* (ISTE 87751).

*A. staticiforme*: GREECE. Antiparos: Ormos Sostis, dune sabbiose, 13 June 1995, *S. Brullo* and *P. Minissale A28* (CAT); ibid., litorale roccioso calcareo, 13 June 1995, *S. Brullo* and *P. Minissale A25* (CAT); Euboea: litorale roccioso di Malakonta, 5 June 1992, *S. Brullo* and *P. Pavone s.n.* (CAT); ibid., rupi calcaree di Akros Kymi, 6 June 1992, *S. Brullo* and *P. Pavone s.n.* (CAT); Kimolos: Goupa, 3 July1994, *S. Brullo and P. Minissale E41* (CAT); ibid., esemplare coltivato, 1 June 1994, *S. Brullo and P. Minissale E41* (CAT); ibid., esemplare coltivato, 27 May 1995, *S. Brullo E41* (CAT); Koufonissi: Harakopou, 30 Agoust 1998, *G. Bartolo* and *S. Brullo* (CAT); ibid., costa di sud-ovest, 30 Agoust 1998, *G. Bartolo* and *S. Brullo s.n.* (CAT); Milos: Mandrakia, esemplare coltivato, 1 June 1994, *S. Brullo E30* (CAT); ibid., Rivari, 1 July 1994, *S. Brullo* and *P. Minissale E35* (CAT); Naxos: Apollonia, scisti costieri, 26 Agoust 1993, *S. Brullo* and *F. Scelsi C11* (CAT); ibid., litorale roccioso di Moutsouna, 11 June 1995, *S. Brullo* and *P. Minissale A17* (CAT); Paros: Agios Fokas, 12 June 1995, *S. Brullo* and *P. Minissale A25* (CAT); ibid., Agia Irini, costa rocciosa calcarea, 13 June 1995, *S. Brullo* and *P. Minissale A26* (CAT); ibid., Chrisi Akti (Drios), 12 June 1995, *S. Brullo* and *P. Minissale A11* (CAT); ibid., Naoussa, Plastiras, litorale roccioso (scisti), 12 June 1993, *S. Brullo* and *P. Minissale A23* (CAT); Schinoussa: Vasilis, 31 Agoust 1998, *G. Bartolo* and *S. Brullo s.n.* Profitis Elias, esemplare coltivato, 27 May 1995, *S. Brullo E11* (CAT).

## 3. Discussion

Within the sect. *Codonoprasum*, *Allium sphaeronixum* shows closest relationships with some other East Mediterranean species belonging to the group of *A. staticiforme*, a species first described from the island of Kimolos (Cyclades archipelago, Greece) [29,30]. The species of this group share some very peculiar features allowing them to be well distinguished from the other taxa of the sect. *Codonoprasum*, so Zahariadi [31] considered it more appropriate to include them in a new section, proposed as *A.* sect. *Phalerea* Zahar. Subsequently, Brullo et al. [12] downgraded it to the rank of a subsection within the sect. *Codonoprasum* and typified by *A. staticiforme*. The most distinctive characters are represented by subglobose inflorescence and are usually dense and compact, subtended by a spathe with two rather small valves, shorter than or subequal to the inflorescence (only rarely longer), perigon white to pink, generally campanulate, small-sized (2–4 mm long), stamen filaments all or at least the inner ones exserted from the perigon, and seeds less than 4 mm long. Based on the literature [7,12,32,33,34,35,36,37,38,39,40], in addition to *A. staticiforme*, many other species sharing these morphological characteristics were described from various territories of the East Mediterranean area, such as *A. flexuosum* d’Urv. from Astypalea island (Greece); *A. myrianthum* Boiss. from West Anatolia; *A. wiedemannianum* Regel from North Anatolia; *A. phalereum* Heldr. and Sartori ex Heldr. from Attica (Greece); *A. weissii* Boiss. from the Cyclades islands (Greece); A. *exiguiflorum* Hayek and Siehe from central–southern Anatolia; *A. thrichocephalum* Nábĕlek and *A. rupicola* Boiss. ex Mouterde from Lebanon; and *A. nazarenum* C.Brullo, Brullo Giusso, and Salmeri from Israel. Currently, many of these names are not recognized as valid distinct species and are often considered critical taxa, regarded as representing synonyms or falling into the intraspecific variability of accepted species [1,35,37,38,40].

**Figure 8:**
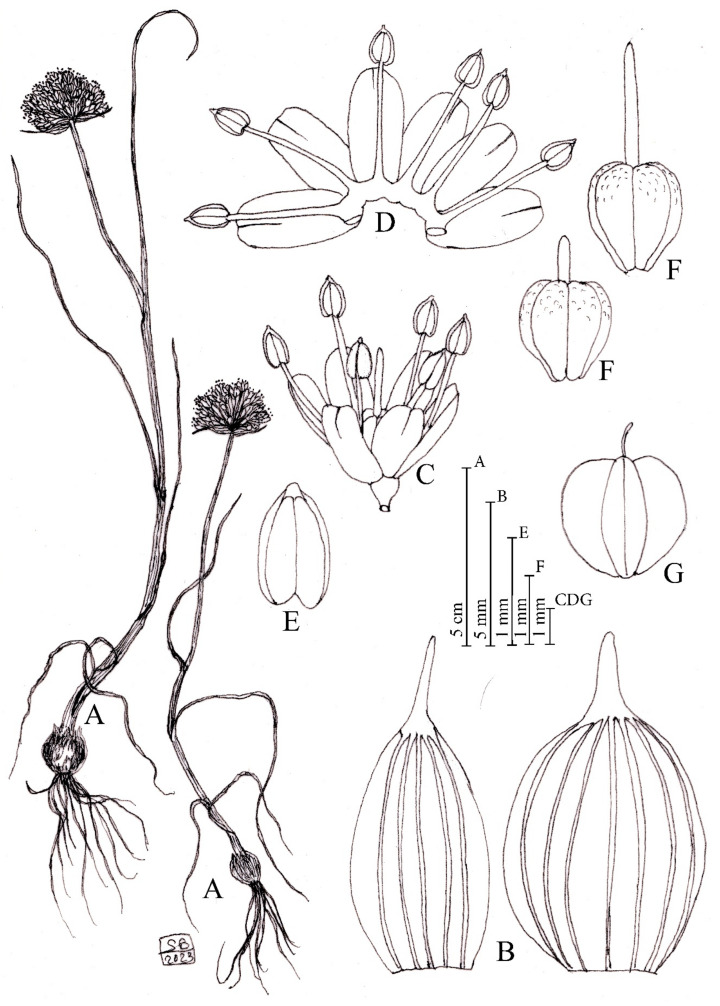
Diagnostic features of *Allium staticiforme*. (A) Habit. (B) Spathe valves. (C) Flowers. (D) Open perigon and stamens. (E) Anther. (F) Ovary. (G). Capsule. Illustration by S. Brullo based on living material from the type locality.

**Figure 9:**
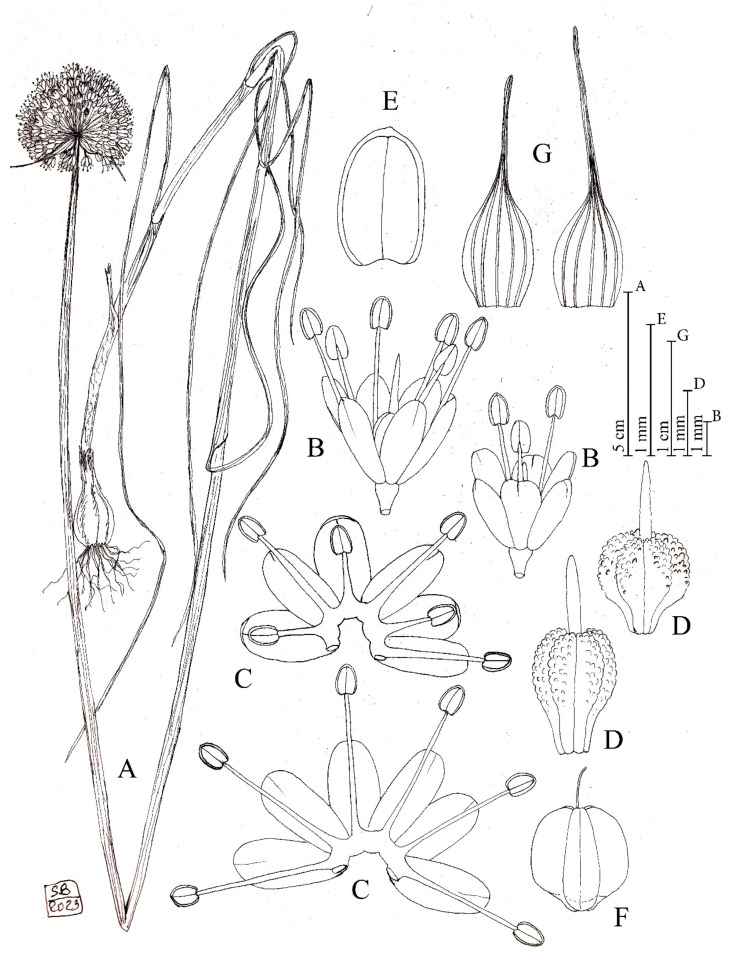
Diagnostic features of *Allium myrianthum*. (A) Habit. (B) Flowers. (C) Open perigon and stamens. (D) Ovary. (E) Anther. (F) Capsule. (G) Spathe valves. Illustration by S. Brullo based on living material from the type locality.

As already highlighted, within the *A. staticiforme* group, *A. sphaeronixum* most resembles *A. staticiforme* (Figure 8) and *A. myrianthum* (Figure 9) for its gross morphology, but many diacritic features allow these species to be well distinguished. The most discriminant characteristics, listed in Table 1, mainly regard the bulb, scape size, number, and size of leaves; size and shape of flower pieces; and capsule and seeds. Specifically, *A. sphaeronixum* differs from the typical populations of *A. staticiforme* in having a bigger size; bulbs without bulbils; fewer leaves which are generally longer and wider; spathe valves unequal and much longer; stamen filaments unequal, the outer ones at first not exserted, shorter annulus; anthers often tinged with purple, shorter and rounded at the apex; ovary shortly stipitate at the base, slightly tuberculate (vs. slightly rugose) and bigger, with a much longer pinkish-white style and a bigger subglobose to globose–ovoid capsule. Other relevant differences regard the leaf cross-sections, with reference to the general outline and many anatomical features, because leaves of *A. sphaeronixum* show a widely fistulous mesophyll with a very limited spongy tissue (vs. filled mesophyll with widely lacunose spongy tissue), larger epidermal cells and a thinner cuticle (cf. Figure 5A), as well as the karyotype structure, as *A. sphaeronixum* has a little more asymmetrical chromosome complement, with one subtelocentric pair and one B-chromosome, which are missing in *A. staticiforme* (cf. Figure 3A,C and Figure 4A,C). Seed coat micromorphology differs as well because *A. sphaeronixum* shows bigger seeds, with testa cells distinct both in size and shape (subpoligonal and nearly isodiametric vs. irregularly polygonal and subisodiametric to elongated), as well as in the micro-sculpturing patterns of both periclinal and anticlinal walls (feebly undulate borders vs. Ω-like).

Despite the general resemblance, significant differences can be clearly detected in comparison with *A. myrianthum*, which diverges from *A. sphaeronixum* due to the bigger size; green and striate scape (vs. glaucous and smooth); more leaves (5–6 vs. 3–4), which are longer but much narrower (40–50% less); bigger inflorescence (3–5 cm vs. 2.5–3 cm), with spathe valves subequal and both shorter than or as long as the inflorescence (vs. unequal, and just the smaller one shorter than inflorescence); perigon white (vs. white suffused with pinkish), with tepals unequal and oblong to linear–oblong, (vs. subequal and oblong-elliptical); white stamen filaments, with anthers yellow and a bit longer; ovary stipitate long at the base, clearly tuberculate and smaller; a white and shorter style; and smaller capsule. Significant differences are also shown in the karyotype structure because, unlike the new species, *A. myrianthum* shows a quite homogeneous chromosome complement only consisting of metacentric pairs (cf. Figure 3A,B and Figure 4A,B). As far as the leaf anatomy is concerned, the cross-section of *A. myrianthum* is clearly different in its smaller size, the semicylindrical outline with very prominent ribs (vs. subcylindrical with inconspicuous ribs), the much larger epidermal cells covered by a well-developed cuticle layer, and the lower number of vascular bundles which all have similar size (vs. numerous, with bigger sized alternating with smaller other ones). Lastly, the seed coat micromorphology further emphasizes the differences between the two species since *A. myrianthum* has smaller seeds, with subrectangular elongated cells (vs. isodiametric) showing a median row of 2–4 smooth verrucae on the periclinal walls (vs. one central large papillate verruca).

In addition to these differences concerning many macro- and micro-morphological, karyological, and anatomical traits, *Allium sphaeronixum* also diversifies in its ecology, which further supports its distinction as a new endemic species. As a matter of fact, while *A. sphaeronixum* grows in mountain habitats associated with maquis and dwarf shrub vegetation, *A. myrianthum* usually occurs in wetlands, where it is a member of marshy communities, and *A. staticiforme* is mainly localized in rocky coastal stands, mixed with dwarf shrub vegetation.

## 4. Materials and Methods

### 4.1. Morphological Study

Plant morphology of the new species was studied using living specimens collected from the *locus classicus* and the other known localities, cultivated 1–2 years both in the Botanical Garden of Catania (Italy) and in the Geophyte Garden in Yalova (Turkey). For taxonomic comparison, living plants of *Allium myrianthum* and *Allium staticiforme*, coming from the respective *loci classici* (Pamukkale in Denizli province, southwestern Turkey, and Kimolos Island in the Cyclades archipelago, South Aegean) and cultivated 1–2 years in the Botanical Garden of Catania, were examined together with several herbarium specimens (AEF, BM, CAT, FI-WEBB, G-BOISS, ISTE, and M). Qualitative and quantitative morphological features were measured and scored on at least 15 fresh samples using a Zeiss Stemi SV11 Apo stereomicroscope at 6–66× magnification. Taxonomic and morphological comparison with the most-related species was based on direct surveys from both fresh and herbarium material. The diagnostic traits of the new species and its two most allied ones are shown in Table 1. Voucher specimens of the new species are deposited in the herbaria CAT and ISTE [41].

### 4.2. Leaf Anatomy

The leaf anatomy was investigated on fresh material from cultivated plants, using blades of minimum-sized and maximum-sized leaves in their optimal vegetative phase from a point 3 cm above the sheaths. Samples were fixed in Carnoy solution (3:1 absolute ethanol: glacial acetic acid), then embedded in paraffin. Leaf cross-sections (ca. 10 µm thick) were double-stained with ruthenium red and light-green yellowish SF and photographed under a Zeiss Axiophot light microscope equipped with a 10 MP digital camera.

### 4.3. Karyology

The karyological analysis was carried out on root tips from cultivated bulbs, pre-treated with 0.3% (*w*/*v*) colchicine water solution for 3 h at room temperature, and then fixed overnight in fresh Farmer’s fixative (3:1 *v*/*v*, absolute ethanol: glacial acetic acid). Root tips were hydrolyzed in 1N HCl at 60 °C for 7 min, washed, and stained with Feulgen for 1 h. Metaphase plates were analyzed and photographed under a Zeiss Axioskop2 light microscope equipped with an Axiocam MRc5 high-resolution digital camera. Karyotype parameters were evaluated from 10 well-spread metaphase plates from 5 individuals; the mean values were used for the karyotype characterization. Metaphase chromosomes were measured using the image analysis system Zeiss Axiovision 4.8, while karyotyping was performed by Cromolab© 1.1 software [42]. The chromosome types were named according to the position of the centromere: r = 1–1.3 (*m*) median, r = 1.3–1.7 (*msm*) median-submedian, r = 1.7–3 (*sm*), r =3–7 (*st*) subterminal [43,44]. All measured karyomorphometric parameters are given in Table 2.

**Table 2:** Karyological features of *A. sphaeronixum* and allied species. Mean values ± standard deviation resulted from 10 good metaphase plates from different individuals of the type locality. Abbreviations: Type = chromosome nomenclature according to Levan et al. [43] and Tzanoudakis [44]; sat = satellited; TCL = total chromosome length; MCL = mean chromosome length; MAR = mean AR; MCI = mean CI; D-value = difference between total L and total S.

*Allium sphaeronixum* (Turkey, Nevşehir)								
Chrom. Number	Long Arm L (µm)	Short Arm S (µm)	Total Length CL = L + S (µm)	Relative Length RL = CL/TKL (%)	*d*-Value L-S (μm)	Arm Ratio AR = L/S	Centromeric Index CI = S/CL × 100	Type
1	5.53 ± 0.3	4.34 ± 0.4	9.88 ± 0.6	7.97 ± 0.3	1.19	1.27	43.98	m
2	5.37 ± 0.4	4.14 ± 0.3	9.51 ± 0.6	7.68 ± 0.3	1.23	1.29	43.53	m
3	5.37 ± 0.7	3.98 ± 0.6	9.34 ± 0.9	7.53 ± 0.3	1.39	1.35	42.54	msm
4	5.33 ± 0.7	3.85 ± 0.5	9.18 ± 1.2	7.40 ± 0.7	1.48	1.38	41.96	msm
5	4.51 ± 0.3	4.18 ± 0.4	8.69 ± 0.7	7.01 ± 0.1	0.33	1.08	48.11	m
6	4.34 ± 0.4	3.98 ± 0.4	8.32 ± 0.7	6.71 ± 0.3	0.37	1.09	47.78	m
7	5.04 ± 0.4	3.41 ± 0.3	8.45 ± 0.6	6.82 ± 0.3	1.63	1.48	40.35	msm
8	4.92 ± 0.2	3.17 ± 0.2	8.09 ± 0.3	6.54 ± 0.2	1.75	1.55	39.21	msm
9	4.10 ± 0.3	3.65 ± 0.2	7.75 ± 0.5	6.25 ± 0.3	0.45	1.12	47.09	m
10	3.93 ± 0.5	3.61 ± 0.1	7.54 ± 0.6	6.25 ± 0.3	0.33	1.09	47.83	m
11	4.07 ± 0.4	3.11 ± 0.7	7.19 ± 0.6	5.80 ± 0.3	0.96	1.31	43.33	msm
12	4.10 ± 0.3	2.91 ± 0.7	7.01 ± 0.5	5.66 ± 0.3	1.19	1.41	41.52	msm
13	4.85 ± 0.5	1.43 ± 0.2	6.29 ± 0.7	5.08 ± 0.5	3.47	3.38	22.82	st^sat^
14	4.59 ± 0.5	1.35 ± 0.3	5.94 ± 0.8	4.80 ± 0.6	3.26	3.39	22.76	st^sat^
15	3.85 ± 0.5	1.52 ± 0.4	5.45 ± 0.6	4.39 ± 0.2	2.46	2.54	27.82	sm^sat^
16	3.69 ± 0.5	1.54 ± 0.3	5.31 ± 0.5	4.28 ± 0.3	2.27	2.39	29.01	sm^sat^
B	2.89 ± 0.2	0.98 ± 0.02	3.82 ± 0.2					
***Allium staticiforme* (Greece, Kimolos Is., Goupa)**								
**Chrom. Number**	**Long Arm** **L (µm)**	**Short Arm** **S (µm)**	**Total Length** **CL = L + S (µm)**	**Relative Length** **RL = CL/TKL (%)**	**d-Value** **L-S (μm)**	**Arm Ratio** **AR = L/S**	**Centromeric Index** **CI = S/CL × 100**	**Type**
1	5.05 ± 0.9	4.95 ± 1.0	10.00 ± 1.9	7.65 ± 0.6	0.11	1.02	49.46	m
2	5.00 ± 0.9	4.84 ± 0.9	9.84 ± 1.8	7.54 ± 0.6	0.16	1.03	49.18	m
3	4.57 ± 0.5	4.41 ± 0.5	8.98 ± 0.9	6.92 ± 0.1	0.16	1.04	49.10	m
4	4.41 ± 0.3	4.30 ± 0.4	8.71 ± 0.7	6.73 ± 0.3	0.11	1.03	49.38	m
5	4.57 ± 0.6	4.14 ± 0.6	8.71 ± 1.2	6.70 ± 0.3	0.43	1.10	47.53	m
6	4.46 ± 0.7	3.87 ± 0.5	8.33 ± 1.4	6.40 ± 0.4	0.59	1.15	46.45	m
7	5.43 ± 0.7	2.80 ± 0.3	8.28 ± 1.0	6.37 ± 0.2	2.69	1.94	33.77	sm^sat^
8	5.38 ± 0.7	2.59 ± 0.6	8.02 ± 1.2	6.17 ± 0.4	2.84	2.07	32.31	sm^sat^
9	4.46 ± 0.5	3.60 ± 0.7	8.06 ± 1.1	6.20± 0.3	0.86	1.24	44.67	m
10	4.46 ± 0.5	3.55 ± 0.6	8.01 ± 0.9	6.17 ± 0.2	0.91	1.26	44.30	m
11	4.62 ± 0.4	2.85 ± 0.4	7.53 ± 0.8	5.80 ± 0.2	1.83	1.62	37.86	msm^sat^
12	4.46 ± 0.4	2.69 ± 0.3	7.20 ± 0.7	5.56 ± 0.4	1.77	1.66	37.31	msm^sat^
13	4.25 ± 0.6	3.23 ± 0.3	7.47 ± 0.9	5.75 ± 0.1	1.02	1.32	43.17	msm
14	4.14 ± 0.5	3.06 ± 0.2	7.20 ± 0.6	5.56 ± 0.2	1.08	1.35	42.54	msm
15	3.66 ± 0.4	3.33 ± 0.2	7.04 ± 0.5	5.44 ± 0.3	0.38	1.10	47.33	m^sat^
16	3.39 ± 0.2	3.15 ± 0.1	6.59 ± 0.2	4.11 ± 0.5	0.29	1.08	47.80	m^sat^
***Allium myrianthum* (SW Turkey, Pamukkale)**								
**Chrom. Number**	**Long Arm** **L (µm)**	**Short Arm** **S (µm)**	**Total Length** **CL = L + S (µm)**	**Relative Length** **RL = CL/TKL (%)**	***d*-Value** **L-S (μm)**	**Arm Ratio** **AR = L/S**	**Centromeric Index** **CI = S/CL × 100**	**Type**
1	4.84 ± 0.6	4.10 ± 0.7	8.93 ± 1.3	7.65 ± 0.3	0.74	1.18	45.87	m
2	4.75 ± 0.5	3.89 ± 0.5	8.64 ± 1.0	7.41 ± 0.1	0.87	1.22	44.97	m
3	4.34 ± 0.3	3.93 ± 0.2	8.28 ± 0.6	7.12 ± 0.3	0.41	1.10	47.52	m
4	4.26 ± 0.2	3.93 ± 0.2	8.20 ± 0.5	7.05 ± 0.4	0.33	1.08	48.00	m
5	4.18 ± 0.1	3.93 ± 0.2	8.11 ± 0.3	6.99 ± 0.4	0.25	1.05	48.48	m
6	4.10 ± 0.2	3.77 ± 0.2	7.87 ± 0.5	6.77 ± 0.3	0.33	1.09	47.92	m
7	4.02 ± 0.6	3.52 ± 0.3	7.54 ± 0.9	6.47 ± 0.1	0.49	1.14	46.74	m
8	3.85 ± 0.6	3.41 ± 0.5	7.26 ± 1.1	6.22 ± 0.3	0.44	1.13	46.95	m
9	3.77 ± 0.7	3.52 ± 0.6	7.30 ± 1.3	6.24 ± 0.4	0.25	1.07	48.31	m
10	3.69 ± 0.6	3.52 ± 0.6	7.21 ± 1.2	6.17 ± 0.3	0.16	1.05	48.86	m
11	3.52 ± 0.3	3.08 ± 0.2	6.61 ± 0.2	5.69 ± 0.5	0.54	1.14	46.65	m^sat^
12	3.61 ± 0.5	2.87 ± 0.3	6.48 ± 0.8	5.55 ± 0.1	0.84	1.26	44.30	m^sat^
13	3.61 ± 0.5	2.95 ± 0.5	6.56 ± 0.9	5.62 ± 0.2	0.75	1.22	45.00	m^sat^
14	3.54 ± 0.5	2.87 ± 0.4	6.41 ± 0.9	5.49 ± 0.2	0.77	1.23	44.76	m^sat^
15	3.03 ± 0.1	2.70 ± 0.3	5.74 ± 0.5	4.93 ± 0.1	0.33	1.12	47.14	m
16	2.87 ± 0.3	2.54 ± 0.3	5.41 ± 0.7	4.64 ± 0.1	0.33	1.13	46.97	m

TCL: 123.93 ± 9.6 µm; MCL: 7.75 ± 1.4 µm; MAR: 1.70 ± 0.8; MCI: 39.35 ± 8.7; D-value: 23.59 µm. Symmetry indices: CV_CL_: 19.29; CV_CI_: 22.21; MCA: 21.18; Stebbins’ Cat.: 2A. TCL: 129.99 ± 15.2 µm; MCL: 8.12 ± 1.0 µm; MAR: 1.31 ± 0.3; MCI: 43.88 ± 5.7; D-value: 14.96 µm. Symmetry indices: CV_CL_: 12.14; CV_CI_: 12.93; MCA: 12.01; Stebbins’ Cat.: 2A. TCL: 116.54 ± 12.5 µm; MCL: 7.28 ± 1.1 µm; MAR: 1.14 ± 0.1; MCI: 46.78 ± 1.4; D-value: 7.43 µm. Symmetry indices: CV_CL_: 14.49; CV_CI_: 3.06; MCA: 6.44; Stebbins’ Cat.: 1A.

### 4.4. Seed Micromorphology

Seed test micromorphology was performed on mature, dry samples coming from herbarium specimens of type locality using a scanning electron microscope (SEM) (Zeiss EVO LS10), according to the protocol reported by Stork et al. [45], while terminology of the seed coat sculpturing followed Barthlott [46,47] and Gontcharova et al. [48].

### 4.5. Geographic Distribution

For the species distribution and conservation status, the GeoCAT software [49], accessed at http://geocat.kew.org/ (accessed on 12 December 2022), was used in order to calculate both the area of occupancy (AOO) and the extent of occurrence (EOO), according to the IUCN Red List criteria [28].

## Figures and Tables

**Table 1:** Morphological comparison between *Allium sphaeronixum* and its closest allied species.

	*A. sphaeronixum*	*A. staticiforme*	*A. myrianthum*
Bulb	not bulbilliferous	bulbilliferous	not bulbilliferous
Scape	20–55 (80) cm high, smooth, glaucous, erect, covered per 1/2 by leaf sheaths	10–25 cm high, smooth, glaucous, erect-ascending, covered for 1/2–1/3 from the leaf sheaths	30–150 cm high, striate, green, covered for 1/3 by the leaf sheaths
Leaves	3–4, blade up to 20 cm long and 2–3 mm wide	5–7, blade up to 15 cm long and 1.5–2.5 mm wide	5–6, blade up to 30 cm long and 1–1.5 mm wide
Spathe	valves unequal, 15–40(–55) mm long, the largest 5 (–7)-nerved, the smallest (3–) 5-nerved, the largest subequal to longer than perigon, the smallest shorter or slightly longer	valves subequal, shorter than perigon, 5–15 mm long, the largest 7-nerved, the smallest 5-nerved	valves subequal, 20–35 mm long, 5-nerved, both shorter than inflorescence to subequal
Inflorescence	(1.5–) 2.5–3 (–4) cm in diameter, pedicels (8–) 10–15 (–20) mm long	1.5–3 cm in diameter, pedicels 5–12 mm long	(2.5) 3–5 cm in diameter, pedicels 10–18 mm long
Tepals	white suffused with pink, subequal, oblong-elliptic, (2.5–) 2.8–3.5 × 1–1.5 (–1.8) mm	white or pale pink, subequal oblong, 2.8–3.5 × 1.2–1.5 mm	white, unequal, inner tepals oblong, 2.5–2.8 (3) × 1.5–1.6 mm, outer tepals linear–oblong, 3–3.2 × 1–1–3 mm
Stamens	filaments white or lightly pinkish-white, unequal, the outer 1.2–2 mm long, first shorter than perigon and then exserted, the inner 3–4 mm long always exserted, with an annulus 0.3–0.4 mm high.	filaments white, all exserted from perigon, 2.8–3.5 mm long, with an annulus 0.5–0.7 mm hight	filaments white, unequal, the outer 1–1.4 mm long, first shorter than perigon and then exserted, the inner 2.8–3.2 mm long always exserted, with an annulus 0.3–0.5 mm high
Anthers	yellow, tinged with purple, oblong, rounded at apex, 0.7–0.9 × 0.5–0.6 mm	yellow, tinged with pink, apiculate at apex, 1.1–1.2 × 0.4–0.5 mm	yellow, oblong, rounded at apex, 1 × 0.6 mm
Ovary	obovoid, shortly stipitate at the base, slightly tuberculate, 1.8–2 × 1.4–1.5 mm	obovoid, long stipitate at the base, slightly rugose, 1.5–1.6 × 1.2–1.3 mm	subglobose-obovoid, long and stipitate at the base, tuberculate, 1.5–1.6 × 1.2–1.5 mm
Style	pinkish-white, 1.9–2 mm long	white, 1–1.7 mm long	white, 1–1.2 mm long
Capsule	subglobose to globose–ovoid, 3.8–4 × 3.8–4 mm	obovoid, 2.5–3.5 × 3.2–3.8 mm	subglobose, 3–3.2 × 3 mm
Seed	3.5–3.7 × 2–2–2 mm	2.9–3.1 × 1.8–1.9 mm	2.9–3.1 × 1.5–1.6 mm

## Data Availability

Data sharing is not applicable to this article.

## Supplementary Materials

The following supporting information can be downloaded at: https://www.mdpi.com/article/10.3390/plants12112074/s1, Figure S1. Color plate with morphological details of *Allium sphaeronixum*. (A,B) General habit. (C) Bulb. (D) Inflorescence. (E) Stamens and pistil (F) Perigon. Figure S2. Color plate of the leaf cross sections of A. sphaeronixum (A1,A2), A. myrianthum (B) and A. staticiforme (C). Figure S3. Details of mesophyll tissues from the leaf cross-sections of *A. sphaeronixum* (A), *A. myrianthum* (B), and *A. staticiforme* (C).
