# Supplementary material for: Allium sphaeronixum (Amaryllidaceae), A New Species from Turkey
Source: Plants (Basel). 2023 May 23;12(11):2074. doi: 10.3390/plants12112074 (PMC10255659; doi:10.3390/plants12112074)
Supplement: Supplementary file 1 [file plants-12-02074-s001.zip › plants-2391926-supplementary.pdf]

Supplementary Materials

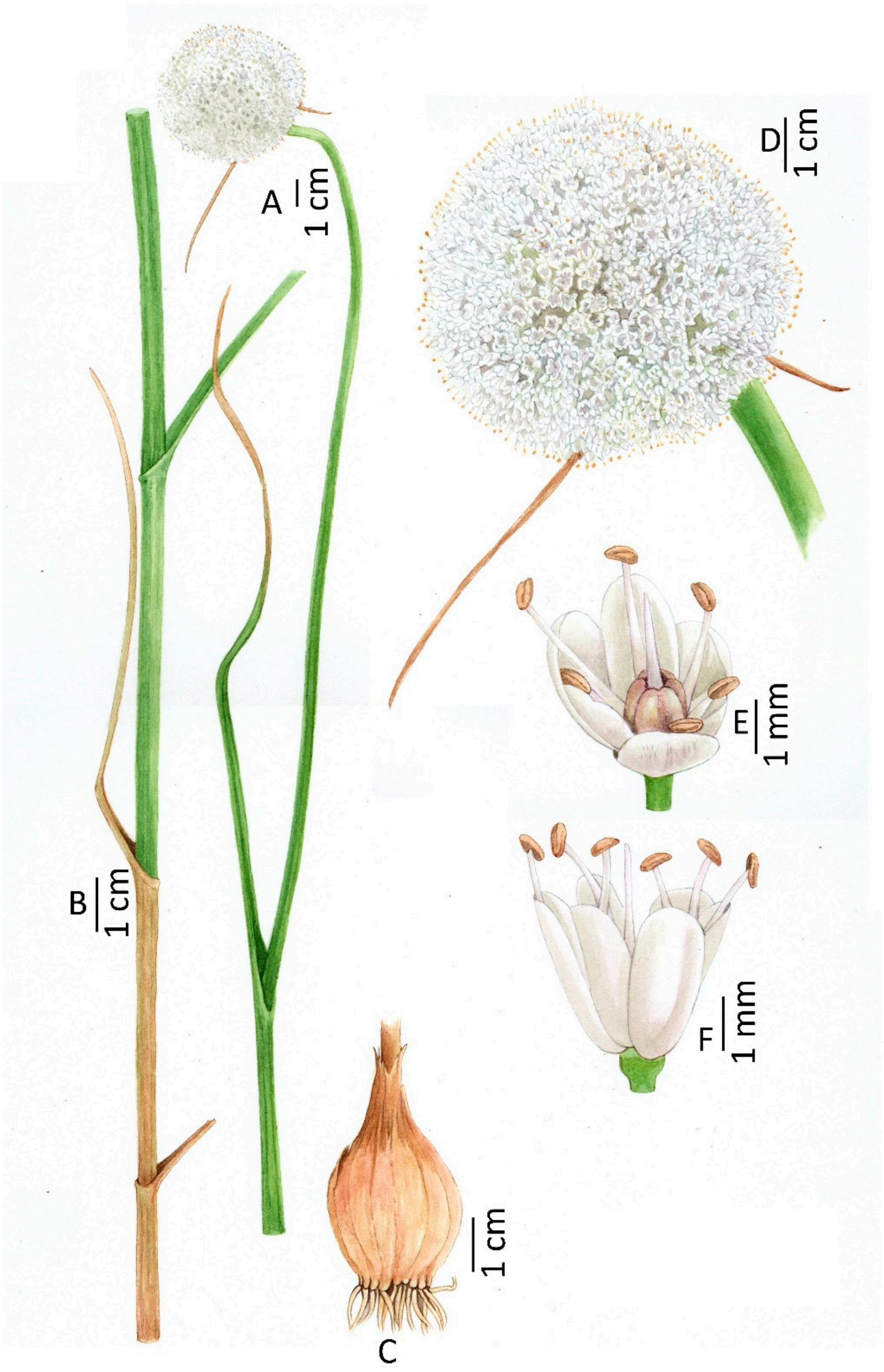

Figure S1. Colour plate with morphological details of *Allium sphaeronixum*. (**A,B**) General habit. (**C**) Bulb. (**D**) Inflorescence. (**E**) Stamens and pistil (**F**) Perigon.

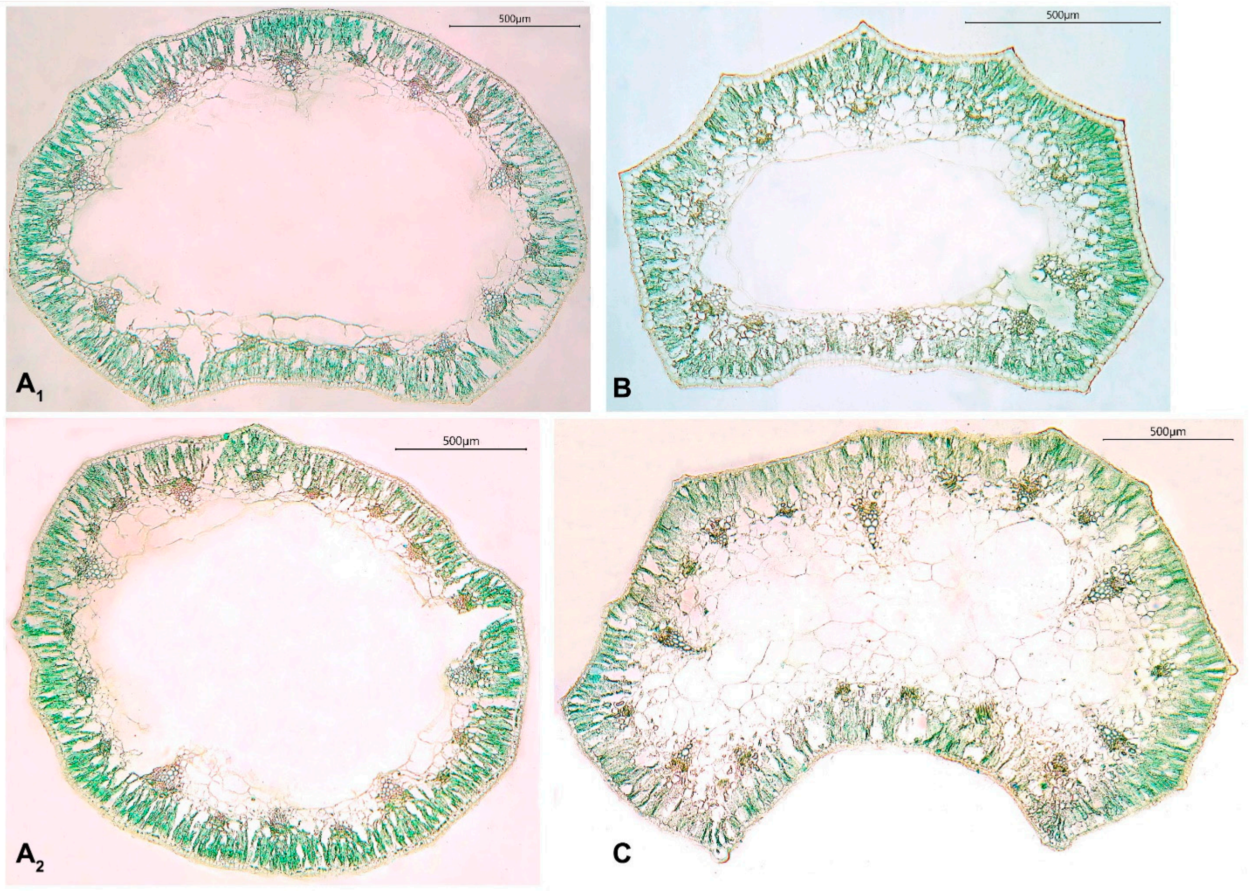

Figure S2. Colour plate of the leaf cross sections of *A. sphaeronixum* **(A_1, A_2)**, *A. myrianthum* **(B)** and *A. staticiforme* **(C)**.

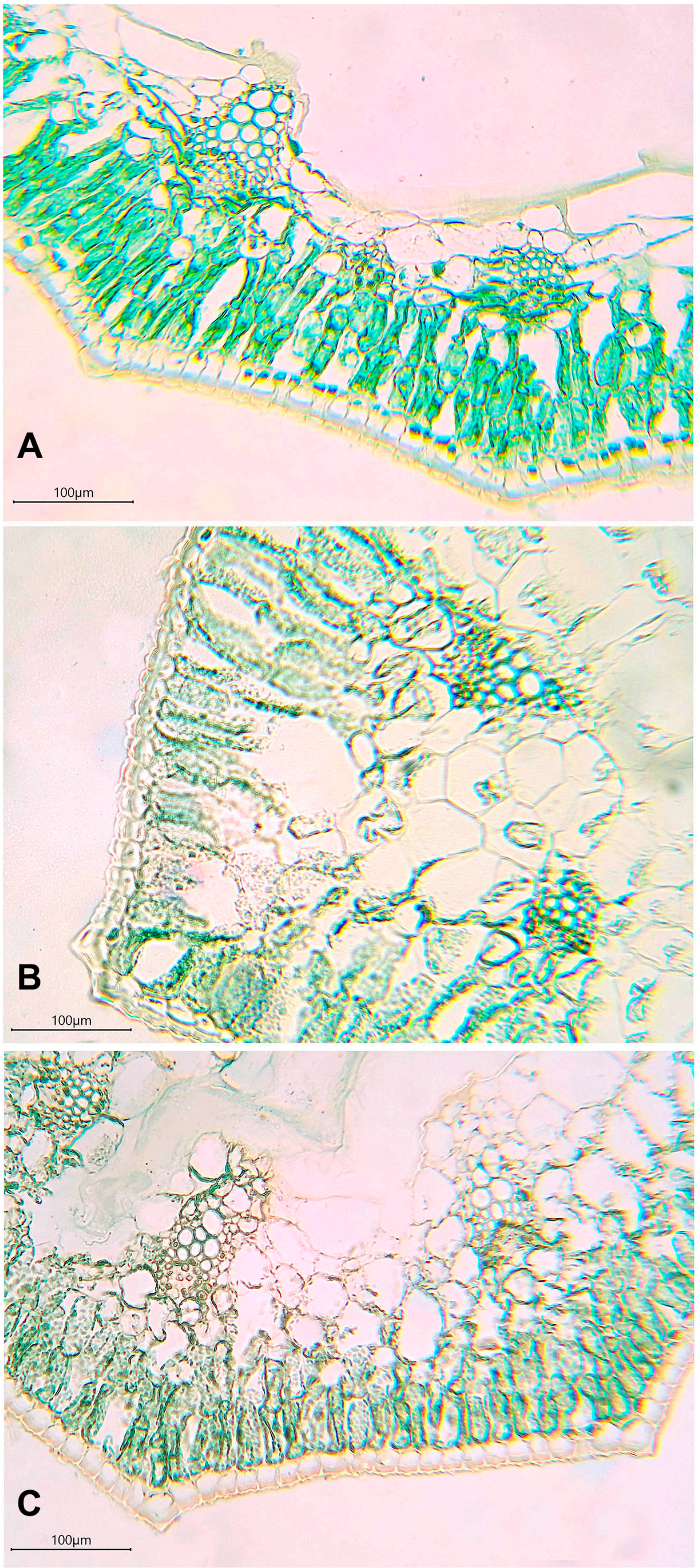

Figure S3. Details of mesophyll tissues from the leaf cross sections of *A. sphaeronixum* **(A)**, *A. myrianthum* **(B)** and *A. staticiforme* **(C)**.
